# Expression of osteopontin-5 splice variant in the mouse primary and metastatic breast cancer cells

**DOI:** 10.1186/s13104-022-06179-w

**Published:** 2022-09-05

**Authors:** Mohammad Kamalabadi-Farahani, Amir Atashi, Zahra Jabbarpour, Seyed Sajjad Aghayan

**Affiliations:** 1 Department of Tissue Engineering, School of Medicine, Shahroud University of Medical Sciences, Shahroud, Iran; 2 Department of Medical Laboratory Sciences, School of Allied Medical Sciences, Shahroud University of Medical Sciences, Shahroud, Iran; 3 Gene Therapy Research Center, Digestive Disease Research Institute, Tehran University of Medical Sciences, Tehran, Iran

**Keywords:** Osteopontin-5, Breast cancer, Splice variants, Alternative splicing, Metastasis

## Abstract

**Objective:**

Osteopontin (OPN) is a well-known glycoprotein involved in numerous pathobiological processes, including cancer. Despite having five splice variants for osteopontin in mice, the main focus of most studies has been on total OPN (tOPN). There are some studies on other splice variants, but the expression of osteopontin-5 (OPN5) has not been addressed in mouse cancer cells. Therefore, this study sought to evaluate OPN5 expression in mouse breast cancer cells.

**Results:**

The expression of OPN5 in primary and metastatic breast cancer cells of mice was confirmed in our study. These findings provided important insights regarding the OPN alternative splicing in mice for the first time. It is concluded that, like other OPN-SVs, OPN5 probably plays an essential role in tumor progression, which requires further investigation in different tumor models.

## Introduction

Osteopontin (OPN), known as secreted phosphoprotein 1 (SPP1), is a popular member of small integrin-binding ligand n-linked glycoproteins in extracellular matrix [1]. OPN exhibits a variety of biological functions under physiological or pathological conditions. The intracellular and extracellular versions of OPN are involved in physiological processes such as mitosis and wound healing, respectively [2–4]. OPN has been extensively studied in cancer. It constitutes the most abundantly secreted phosphoprotein in breast cancer and supports invasive behavior. Hence, it is a biomarker for breast cancer aggressiveness and prognosis (the abundance of OPN correlates negatively with survival) [5, 6].

The human gene SPP1 encodes OPN located on the long arm of chromosome 4 and its murine counterpart Spp1 located on the long arm of chromosome 5 [7, 8]. Human and mouse OPN cDNA samples share a high degree of sequence homology with human and mouse genes consisting of seven exons extending over 8 and 7 kb, respectively [9, 10]. Alternative splicing has a critical importance for the functional diversity of proteins, especially in cancer cells [11]. For OPN, since 1995, alternative splicing has been reported in glioma cancer cells [12]. Alternative splicing of the OPN transcript produces five isoforms in human [13] deposited in public databases. Among them, Osteopontin-a (OPNa), Osteopontin-b (OPNb), and Osteopontin-c (OPNc) are the best functionally characterized isoforms to date and have been consistently reported [14]. These three OPN-SV have been extensively studied, focusing on their expression patterns and functional roles in cancer cells.

Apart from OPNa, OPNb, and OPNc, recent research depict OPN4, and OPN5 as a new splice variants of OPN that annotated in NCBI, ENA, and UniProt [15]. In a recent work on human OPN-SV the expression profile of osteopontin-4 (OPN4) and osteopontin-5 (OPN5) has been addressed in distinct cancer cell lines. OPN4 and OPN5 transcripts displayed co-expression in most tested cell lines [16]. Also, OPN5 in human esophageal adenocarcinoma was described by Lin et al. [17].

Studies emphasize that alternative splicing of osteopontin is not known to occur in mice, and there is no report regarding the investigation of OPN5 in any tumor types or cancer cells in the mouse to the extent of our knowledge [18]. In this regard, the present study was designed to investigate the expression of OPN5 in mouse breast cancer cells and its expression level compared to other tOPN isoforms.

## Main text

### Materials and methods

#### Cell culture

The murine mammary carcinoma cell line 4T1 was obtained from the cell bank of Pasteur Institute of Iran (C604) and cultivated in high glucose Dulbecco’s Modified Eagle’s Medium (DMEM) containing 10% FBS (fetal bovine serum) and 2% Penicillin–Streptomycin (all from Gibco, USA). The cells incubated in 37 °C with 95% air and 5% of carbon dioxide (CO_2_).

#### Mammary tumor induction and isolation of primary and highly metastatic breast cancer cells

Induction of mammary tumor in BALB/c mice and isolation of primary and metastatic tumor cells, were performed as described in our previous work [19, 20]. The Ethics Committee of Shahroud University of Medical Sciences approved this study for ethics in animal research (registration number: IR.SHMU.REC.1400.265). The isolated cells were cultured in a DMEM with 10% FBS, 100 U/ml Penicillin, and 100 ug/ml Streptomycin (all from Gibco, USA). Ultimately, the cells were incubated at 37 °C in 5% CO_2_ and passaged two times.

#### Real-time qRT-PCR assay

Primary and metastatic tumor cells (1 × 10^4^) were seeded in a 24-well plate. Total RNA extraction, cDNA synthesis and Real-time PCR procedure were preformed similar to our previous works [20]. Briefly after 48 h, total RNA was extracted from the seeded cells using Trizol reagent. The extracted RNA's quality, yield, and size were analyzed using spectrophotometry and electrophoresis. The first-strand cDNA synthesis was performed using a reverse transcription system (Easy cDNA Synthesis Kit for RNA or mRNA to cDNA—pars tous). A Real-time PCR procedure was executed based on the 1 ul cDNA in all samples. According to the manufacturer's instruction, quantization of all gene transcripts was done by SYBR Green Real-time PCR Master Mix (Amplicon A/S, Denmark) using StepOnePlus™ Real-Time PCR System. The amplification procedure was as follows: 1 cycle of 95 °C for 15 °min, 40 cycles of 95 °C for 30 s, 60 °C for 30 s, and 72 °C for 30 s. The exact mRNA expression was normalized to the expression level of GAPDH. Gene expression of each target was calculated by using the 1/ΔCT method. GAPDH amplification was used as normalization controls for OPN transcription level evaluation and the RT-qPCR. The delta CT value was calculated by applying the following calculation: (CT of OPN (tOPN or OPN5)) − (CT of housekeeping gene).

The used primers are as follows:

For tOPN, Forward 5′-GGATGAATCTGACGAATCTCAC-3′, Reverse 5′- CCTTAGACTCACCGCTCTTC-3′;

For OPN5, Forward 5′-TGGTGGTGATCTAGTGGTG-3′, Reverse 5′- CATGGTCGTAGTTAGTCCTG-3′;

For GADPH, Forward 5′-CCTGGAGAAACCTGCCAAGTA-3′, Reverse 5′-GGCATCGAAGGTGGAAGAGT-3′.

#### Statistical analysis

Results are expressed as the mean ± standard deviation. Data were analyzed by GraphPad Prism statistical software 6.0 (GraphPad Software, La Jolla, CA, USA) using Paired Samples t-test. P < 0.05 was considered statistically significant.

### Results

#### Isolation of primary and metastatic tumor cells

**Fig. 1:**
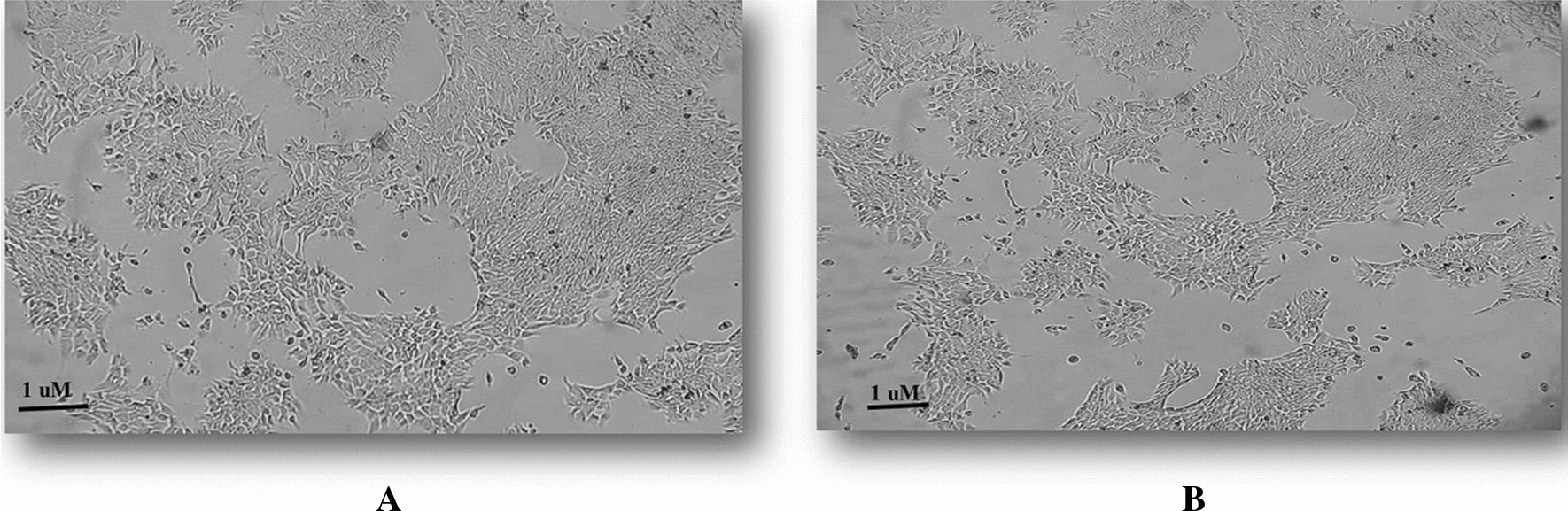
Primary and metastatic cancer cells. **A** Highly metastatic breast cancer cells. **B** Primary breast cancer cells

Due to multiple passages and manipulations, most breast cancer cell lines have changed their function and genomes [21]. So, we decided to use the primary and highly metastatic tumor cells that were isolated from cancerous mice tissues. We properly extracted primary and metastatic tumor cells from subcutaneous primary tumor and lung of cancerous mice, respectively. The metastatic tumor cells in the lung after primary isolation, form colonies in the culture medium. Due to the high rate of growth and proliferation, the tumor cells in these colonies are purified after three passages. These tumor cells are called lung metastatic tumor cells or 4T1L (Fig. 1A) while tumor cells that are obtained in the same way, from the original tissue of the tumor, are primary tumor cells called 4T1T (Fig. 1B).

#### OPN has five splice variants and four isoforms in mouse

**Fig. 2:**
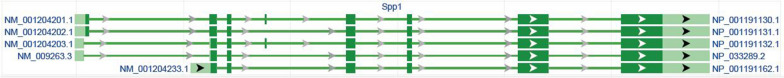
Alternative splicing of OPN in mouse. Transcript variants of OPN in mice (OPN-SV) have been deposited in public databases such as NCBI. In mouse OPN have 5 splice variants that code 4 very similar proteins

Although OPN has five splice variants (Fig. 2) and four isoforms in mice, total OPN (tOPN) is the main focus of most studies of OPN in cancer. Hence, in this study, we used two primer sets; the first primer set covers all splice variants of OPN, and the second set only covers OPN5.

#### Expression of OPN5 was confirmed in primary and metastatic tumor cells

**Fig. 3:**
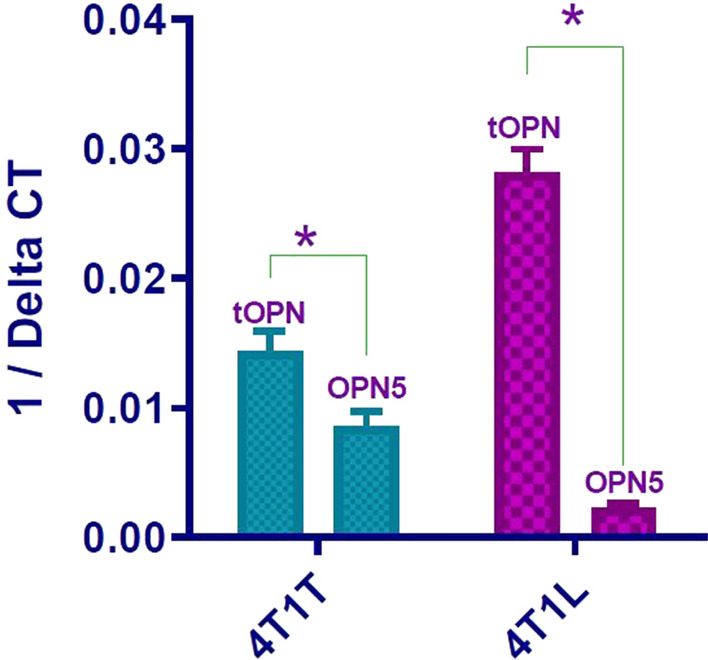
Expression of OPN5 in primary and metastatic tumor cells. According to Real-Time PCR result, expression of OPN5 in mouse breast cancer cells was revealed for the first time. In 4T1T 80% of tOPN expression belongs to OPN5. In 4T1L OPN5 constitute only 10 of tOPN expression. All results are expressed as mean ± SD from at least three independent experiments analyzed by a Two-tailed T-test (* P < 0.001)

The expression of tOPN and OPN5 was analyzed in the primary and metastatic tumor cells. As shown in Fig. 3, both in 4T1T and 4T1L OPN5 were expressed. In 4T1T 80% of tOPN expression belongs to OPN5 but in in 4T1L OPN5 constitute only 10 of tOPN expression.

## Discussion

The critical role of OPN in breast cancer progression and metastasis has been confirmed in numerous studie [22]. Although OPN has five splice variants in mice, total OPN (tOPN) is the main focus of most studies of OPN in cancer [23]. Our study revealed the expression of OPN5 in mouse breast cancer cells for the first time. According to our results, about 60% of total tOPN expression belongs to OPN5 in breast cancer cells isolated from mice. These findings provided important insights regarding the OPN alternative splicing in mice for the first time. It is concluded that, like other OPN-SV, OPN5 probably plays an essential role in tumor progression, which requires further investigation in different tumor models.

In the only study on OPN-SV in mice, the authors emphasized that alternative splicing of osteopontin is not known to occur in mice. They have not detected more than one message in various murine tissues by RT–PCR. They indicated that mouse models might have limitations for investigating osteopontin in cancer due to the restrictions in structural diversity of OPN [18]. In contradiction with this report, in our study with two primer sets, first, that cover all splice variants of OPN and second that only cover OPN5, we detect alternative splicing of OPN in the mouse.

In some of human solid tumors, the roles of OPN-SV, including OPN4 and OPN5 have been investigated. In 2015, the expression of OPN4 and OPN5 were detected in esophageal adenocarcinoma by Lin etal. In their study, the expression patterns of OPN-SV were investigated only in EACs tissue samples [17]. Considering that we used mouse tumor cells but not tissue samples in our study, mouse breast tumor tissue may exhibit a distinct expression pattern for these OPN-SV, which should be further investigated.

In human cancer cell lines, results indicated that OPN4 and OPN5 transcripts displayed co-expressed in most have been assessed cell lines except for MDA-MB-231 and MCF-7, which only express OPN5 [16]. In compliance with this research, we detect the expression of OPN5 in mouse breast tumor cells. It might be an essential clue for the pivotal role of OPN5 in breast cancer. The point that makes it necessary for further research in this regard. We analyze OPN5 expression at the transcription level.

In a recent study on human skin cancer tissue and cell line, researcher found that OPN5 expression was higher than OPNb and OPNc in normal skin. In addition, in nonmelanoma skin cancer, OPN5 expression was higher than OPNc. This study also emphasizes the pivotal role of OPN5 in the skin cancer [24].

There are some challenges regarding protein analysis of OPN5 because we have no specific antibodies against this splice variant [25]. These restrictions result in most papers studying OPN-SV evaluating their roles in cancer cells using isoforms specific oligonucleotide pairs at the transcriptional levels.

## Conclusion

In conclusion, our study provided strong evidence that OPN5 splice variants are widely expressed in mouse breast cancer cells. This OPN splice variant constitute a large part of the overall expression of the OPN in these cells. Moreover, once OPN5 can be expressed in lower or higher levels regarding the remaining OPN-SV, these expression profiles provide some clues regarding their functional roles of this variant in tumor progression, which should be further investigated. Furthermore, the functional roles of OPN5 in distinct aspects of tumor progression and their interaction with another OPN-SV remain unclear. Further research is also needed to determine whether the expression of OPN5 could be used as a biomarker in breast cancer.

## Limitation

Designing a specific primer set for different splice variants of the osteopontin gene is the major limitation of this project because these splice variants have a very high overlap.

## Data Availability

The datasets used and/or analyzed during the current study available from the corresponding author on reasonable request.

## Abbreviations

OPN: Osteopontin
tOPN: Total OPN
OPN5: Osteopontin-5
TNBC: Triple-negative breast cancer
OPN-SVs: OPN splice variants
SPP1: Secreted phosphoprotein 1
OPNa: Osteopontin-a
OPNb: Osteopontin-b
OPNc: Osteopontin-c
EAC: Esophageal adenocarcinoma
